# Optimal *ex vivo *expansion of neutrophils from PBSC CD34^+ ^cells by a combination of SCF, Flt3-L and G-CSF and its inhibition by further addition of TPO

**DOI:** 10.1186/1479-5876-5-53

**Published:** 2007-10-30

**Authors:** Olga Tura, G Robin Barclay, Huw Roddie, John Davies, Marc L Turner

**Affiliations:** 1 SNBTS Adult Cell Therapy Group, Scottish Centre for Regenerative Medicine, University of Edinburgh School of Clinical Sciences, The Chancellor's Building, 49 Little France Crescent, Edinburgh EH16 4SB, UK; 2 NHS Lothian University Hospitals Division, Department of Haematology, Western General Hospital, Edinburgh EH4 2XU, UK

## Abstract

**Background:**

Autologous mobilised peripheral blood stem cell (PBSC) transplantation is now a standard approach in the treatment of haematological diseases to reconstitute haematopoiesis following myeloablative chemotherapy. However, there remains a period of severe neutropenia and thrombocytopenia before haematopoietic reconstitution is achieved. *Ex vivo *expanded PBSC have been employed as an adjunct to unmanipulated HSC transplantation, but have tended to be produced using complex cytokine mixtures aimed at multilineage (neutrophil and megakaryocyte) progenitor expansion. These have been reported to reduce or abrogate neutropenia but have little major effect on thrombocytopenia. Selective megakaryocyte expansion has been to date ineffective in reducing thrombocytopenia. This study was implemented to evaluate neutrophil specific rather than multilineage *ex vivo *expansion of PBSC for specifically focusing on reduction or abrogation of neutropenia.

**Methods:**

CD34^+ ^cells (PBSC) were enriched from peripheral blood mononuclear cells following G-CSF-mobilisation and cultured with different permutations of cytokines to determine optimal cytokine combinations and doses for expansion and functional differentiation and maturation of neutrophils and their progenitors. Results were assessed by cell number, morphology, phenotype and function.

**Results:**

A simple cytokine combination, SCF + Flt3-L + G-CSF, synergised to optimally expand and mature neutrophil progenitors assessed by cell number, phenotype, morphology and function (superoxide respiratory burst measured by chemiluminescence). G-CSF appears mandatory for functional maturation. Addition of other commonly employed cytokines, IL-3 and IL-6, had no demonstrable additive effect on numbers or function compared to this optimal combination. Addition of TPO, commonly included in multilineage progenitor expansion for development of megakaryocytes, reduced the maturation of neutrophil progenitors as assessed by number, morphology and function (respiratory burst activity).

**Conclusion:**

Given that platelet transfusion support is available for autologous PBSC transplantation but granulocyte transfusion is generally lacking, and that multilineage expanded PBSC do not reduce thrombocytopenia, we suggest that instead of multilineage expansion selective neutrophil expansion based on this relatively simple cytokine combination might be prioritized for development for clinical use as an adjunct to unmanipulated PBSC transplantation to reduce or abrogate post-transplant neutropenia.

## Background

Restoration of haematopoiesis by autologous transplantation of haematopoietic stem cells (HSC) following myeloablative chemotherapy has become standard treatment for a number of malignant disorders. Use of cytokine mobilised peripheral blood stem cells (PBSC) has generally reduced the period of post transplant neutropenia and thrombocytopenia compared to use of bone marrow HSC. Identification of mobilised PBSC by CD34^+ ^expression and collection by leukapheresis has demonstrated that the period of neutropenia and thrombocytopenia may be shortened by increasing the dose of CD34^+ ^cells transplanted. However there still remains a period of clinically significant neutropenia and thrombocytopenia which cannot be reduced by increasing CD34^+ ^cell doses. This is probably related to the minimum time required for adequate post transplant expansion and maturation of relevant HSC *in vivo*. Several groups have therefore investigated *ex vivo *expansion of PBSC prior to transplantation, to attempt to further reduce or abrogate post transplant neutropenia and thrombocytopenia, and which has been the subject of a number of recent commentaries and reviews[1-5].

CD34^+ ^cells are heterogeneous and include primitive multipotent stem cells and more mature lineage-committed haematopoietic progenitors. When purified they can, by themselves, restore haematopoiesis and therefore contain all necessary cell types although these cannot readily be discriminated by phenotype[6-9]. The availability of recombinant cytokines has allowed investigation of the role of different cytokines in driving proliferation and maturation of CD34^+ ^cells with different haematopoietic potential, and investigation of the use of different combinations of cytokines for expansion of HSC for different clinical objectives. To date, none of these *ex vivo *protocols has been adopted for routine clinical use although some have demonstrated clinical potential, especially with regard to reduction of neutropenia[10-15]. Most do not compare favourably on a cost-benefit basis to conventional support for HSC transplantation such as transfusion of blood or blood components. However, support for neutropenia by allogeneic donor granulocyte transfusion is not routinely available[16-19], unlike platelet transfusion support for thrombocytopenia, and neutropenic patients remain at risk from life-threatening infection. In this context it may be informative to examine specific expansion of neutrophil precursors from autologous PBSC, as an intended adjunct to unmanipulated autologous PBSC transplantation, to determine whether a simple cytokine combination might achieve this when expansion of other HSC elements such as long term repopulating cells or megakaryocyte precursors is not required.

A number of studies have examined *ex vivo *expansion of the more mature progenitor component of PBSC thought to be responsible for short term reconstitution of neutrophils and platelets, where such manipulated cells would be given together with standard unmanipulated PBSC which ensure long term sustained haematopoiesis. In most cases the *ex vivo *protocols have aimed to achieve simultaneous expansion of both neutrophil and megakaryocyte precursors to address the dual problems of neutropenia and thrombocytopenia. In many such instances the combinations of cytokines used are complex, and often there is no systematic substantiation of the requirement for each cytokine in the protocol. The simplest protocols aimed at simultaneous neutrophil and megakaryocyte precursor expansion from mobilised autologous CD34^+ ^cells have employed only three cytokines, namely stem cell factor (SCF) as a proliferative stimulus together with granulocyte colony stimulating factor (G-CSF) to drive neutrophil maturation and thrombopoietin (TPO) to drive megakaryocyte maturation[10-13,15]. These *ex vivo *expanded PBSC have reduced neutropenia when used to supplement unmanipulated autologous PBSC infusions, but with few exceptions have not demonstrated any significant effect on thrombocytopenia. More complex general expansion protocols, incorporating or derived from protocols intended to expand multipotent long-term reconstituting HSC, have not been tested clinically. Expansion protocols aimed specifically only at megakaryocyte generation are safe but have so far not achieved significant reduction of thrombocytopenia when applied clinically[20-22]. It therefore seems that while reduction of thrombocytopenia remains elusive for autologous PBSC engraftment, *ex vivo *expanded PBSC as a supplement to unmanipulated PBSC might provide feasible reduction of neutropenia if convenient and cost effective expansion protocols can be determined.

We have investigated optimal cytokine combinations for *ex vivo *expansion of neutrophil precursors by examining a group of cytokines employed in a number of reports of *ex vivo *expansion of PBSC. We initially examined cytokines alone and in combination for maximal proliferative expansion of PBSC CD34^+ ^cells, then we examined the supplementary cytokine combinations required for maturation of the expanded cells by assessing expression of aspects of neutrophil morphology, phenotype and function.

## Methods

### Purification of CD34^+ ^cells

Venous blood samples (10 mL) rich in HSC were collected in heparin from patients immediately following cell-separator leukapheresis collection of G-CSF mobilised PBSC for autologous transplant. Mononuclear cells were separated by buoyant density centrifugation over Ficoll-Histopaque (1.077 g/ml; Sigma Diagnostics, UK) and washed twice in phosphate buffered saline (PBS), and counted. The CD34^+ ^fraction was enriched using either negative selection (Rosette-Sep, Stem Cell Technologies, UK) or positive selection (MACS CD34 isolation Kit; Miltenyi Biotec, Surrey, UK), according to the manufacturers instructions. The efficiency of the purification of CD34^+ ^cells was verified by flow cytometry phenotyping analysis.

### Flow cytometry analysis

Whole blood, CD34^+ ^enriched cells, or cells after culture expansion were phenotyped by flow cytometry. Cells were stained with PerCP-conjugated anti-human CD45 and PE-conjugated anti-human CD34, fixed and gated for CD45^+ ^CD34^+ ^cells with a low side scatter, according to the ISHAGE CD34 enumeration protocol[23]. Unmanipulated and cultured cells were also analysed for expression of surface markers specific for subpopulations of haematopoietic cells. The MAbs included anti-CD34-PE and CD41-FITC (Becton Dickinson, Oxford, UK) and anti-CD16b-FITC (Immunotech, UK). Appropriate isotype negative controls were compared with unstained samples, and since these did not differ unstained samples were used to establish positive stain boundaries. 50–100 ul of the sample were stained with the appropriate antibodies for 30 minutes in the dark, any erythrocytes were lysed, and cells were washed with PBS at 200 g. The cells were resuspended in Cell Fix (Becton Dickenson) and events were collected by a FACS Calibur System equipped with CellQuest software (Becton Dickenson) and analysed offline using FCS Express software (De Novo Software[24])

### MPO intracytoplasmic stain

Anti-myeloperoxidase, MPO-7 MAb (Dako Cytomation Ltd, Cambridgeshire, UK) strongly labels the cytoplasm of mature and immature neutrophils. Monocytes are weakly positive while eosinophils are unreactive. 50 ul of sample was stained with anti-CD34-PE and anti-CD45-PerCP, as above. The sample was fixed using 100 ul DAKO Intrastain Reagent A (fixation), and incubated and washed with PBS. 100 ul of the DAKO Intrastain Reagent B (permeabilisation) and 5–10 ul of the anti-MPO-FITC cytoplasmic antibody was added, mixed, washed and resuspended in an appropriate fluid for flow cytometry analysis.

### Ex vivo expansion cultures

Iscove's modified Dulbecco's medium (IMDM) (Invitrogen, Paisley, UK) supplemented with 10% FCS (Sigma, UK) and 1% antibiotic (Pen/Strep, Invitrogen) was used for cultures. CD34^+^-enriched PBSC (2 × 10^5 ^total cells per ml) were plated in 24-well plates in 1 ml medium with or without various cytokines. The following recombinant purified human cytokines were used in these studies: flt-3 ligand (Flt3-L), stem cell factor (SCF), interleukin 3 (IL-3), interleukin 6 (IL-6) and thrombopoietin (TPO) from PeproTech Ec Ltd, London, UK. Recombinant human granulocyte colony-stimulating factor (G-CSF; lenograstim) was a gift from Chugai Pharma, UK. Cells were cultured in 100 ng/ml SCF, 10 ng/ml Flt3-L unless otherwise stated, with or without 100 ng/ml G-CSF and with or without 100 ng/ml TPO. Cells were cultured in a fully humidified atmosphere of 5%CO_2 _in air, at 37C for 14 days. Following vigorous pipetting aliquots were removed for cell counts, differential morphology, flow cytometry analysis and colony assays. CD34^+ ^numbers were calculated from total cell numbers and CD34^+ ^proportions: CD34^+ ^expansion was expressed as a "fold" expansion over starting CD34^+ ^numbers, where fold expansion is the final number of cells after expansion divided by the initial CD34^+ ^numbers before culture.

### Giemsa staining and morphological analysis

The morphology of the cells was determined using a standard Wright-Giemsa-stained cytospin preparations (Thermo Shandon, UK). Neutrophils were scored according to their maturity appearance on advice from experienced clinical haematologists, and was principally based on the complexity of their nuclear morphology.

### Neutrophil chemiluminescence assay

Bioluminescence assays were carried out to measure the superoxide respiratory burst activity of CD34^+ ^derived cells. This was measured by detecting luminol-amplified chemiluminescence responses to phorbol myristate acetate (PMA) (Sigma). In a 96-well opaque microplates, 100 ul of isolated mature peripheral blood neutrophils or expanded cultured progenitors at 0.5 × 10^6 ^cells/ml in IMDM without phenol red and without FCS were plated with 100 ul of luminol (1 mM) (Sigma) and 100 ul of PMA (1 ug/ml final concentration), and the emitted light activity (relative light units, rlu) was measured on a microplate luminometer (Labsystems Luminoskan) at 37C every 3 minutes over 90 minutes.

### Colony assays

Colony forming cells (CFC) were monitored by their growth in methylcellulose (MC) medium. 50 ul of cells were plated at 1.5 × 10^4 ^cells in 500 ul of MC containing cytokines (MethoCult H4435; Stem Cell Technologies, UK). Cells were plated in 24 well-plates. Colonies (groups of >100 cells) formed were scored according to appearance and counted after 14 days. Due to low cell numbers of CD34^+ ^enriched cells (from 10 mL blood samples), day 0 CFC were measured on mononuclear cell isolates before CD34^+ ^enrichment whereas day 14 CFC were measured on cultured CD34^+ ^enriched cells, which by day 14 of culture are all CD34^+ ^in the cytokine combinations studied. The results are expressed for each colony type as a percent of total CFC and are intended for qualitative interpretation, but for approximate quantitative guidance are also given as colony numbers per thousand cells with CD34^+ ^cell proportions and fold increase.

## Results

### CD34^+ ^cell enrichment

CD34^+ ^cells were in general enriched to a purity of around 15–30% using the Rosette-Sep (negative-selection) method or 60–90% using the Miltenyi (positive-selection) method. Better total yields of CD34^+ ^cells were obtained with the negative-selection method which was used in most cases, but similar outcomes were obtained with CD34^+ ^cells enriched by the positive selection method and these were included in the results.

### PBSC expansion

**Figure 1:**
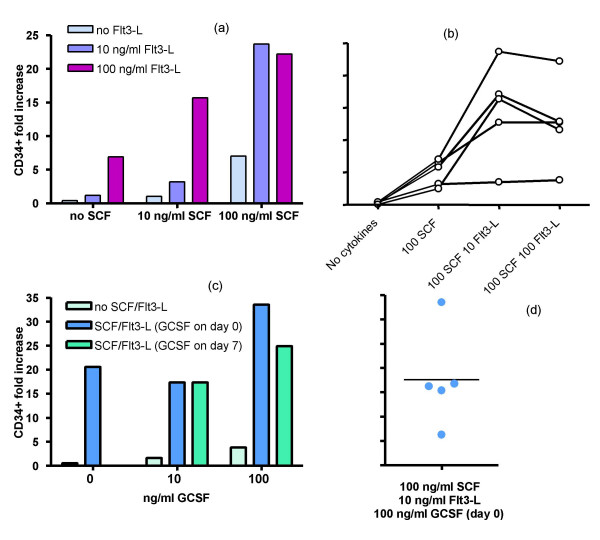
**Increase in CD34^+ ^cells in 14-day cultures with different doses of SCF, Flt3-L and G-CSF alone and in combination**. (a) Responses of a representative patient's CD34^+ ^cells to different combinations of SCF and Flt3-L showing the greatest increase with the combination of 100 ng/ml SCF and 10 ng/ml Flt3-L. (b) Responses of five different patients showing that in general 10 ng/ml Flt3-L gives a greater response than 100 ng/ml Flt3-L in combination with 100 ng/ml SCF. (c) Responses of a representative patient's CD34^+ ^cells to 100 ng/ml SCF + 10 ng/ml Flt3-L added at day zero, alone or with 100 ng/ml G-CSF added at day zero or day 7. (d) Range of five patients' responses to 100 ng/ml SCF + 10 ng/ml Flt3-L + 100 ng/ml G-CSF (these are a different five patients from those shown in b).

We examined doses and combinations of the cytokines to expand CD34^+ ^PBSC in 14-day cultures. Initially SCF and Flt3-L were examined alone and in combination (Figure 1a &1b). SCF and Flt3-L, each at 100 ng/ml, gave similar expansion when used alone, but appeared to synergise when used in combination in that the expansion (up to over 20-fold in some cases) exceeded the summed expansion with either alone. In general the combination of SCF (100 ng/ml) with 10 ng/ml Flt3-L was superior to the combination of SCF with 100 ng/ml Flt3-L (Fig 1b), but this did not reach significance (p = 0.156, Wilcoxon signed rank test) in the five patients studied. The combination of 100 ng/ml SCF with 10 ng/ml Flt3-L was adopted for further studies with other cytokines.

In a series of tests, 100 ng/ml G-CSF was superior to 10 ng/ml G-CSF (not shown). Addition of 100 ng/ml of G-CSF to the SCF/Flt3-L combination gave a further synergistic expansion of the enriched CD34^+ ^cells (to up to over 30-fold in some cases) (Figure 1c and 1d), whereas G-CSF alone showed low expansion potential. The addition of G-CSF to the SCF/Flt3-L combination at the start of cultures (day 0) was in general superior to the addition of G-CSF midway (day 7) through cultures, which implies that G-CSF synergises with SCF/Flt3-L during early proliferative expansion rather than acting only to differentiate and mature cells expanded by SCF/Flt3-L alone. In 9 patients where SCF/Flt3-L alone or in combination with G-CSF at day 0 has been tested there is a wide range of expansion responses (3 to 34 fold for the latter combination), but in all cases there was superior expansion with SCF/Flt3-L/G-CSF than with SCF/Flt3-L (p = 0.0019, Wilcoxon signed rank test). The comparison of expansion by addition of G-CSF at either day 0 or day 7 of the 14 day cultures did not reach significance (p = 0.062, Wilcoxon signed rank test).

Other cytokines which were tested in combination with optimal SCF/Flt3-L/G-CSF were IL-3, IL-6 and TPO. Either IL-3 or IL-6 alone slightly lowered the optimal SCF/Flt3-L/G-CSF expansion, but not significantly, whereas together they slightly enhanced the optimal SCF/Flt3-L/G-CSF expansion, but not significantly (not shown). Since these effects were not overt, and for the sake of simplicity for any implementation of clinical protocols for cell expansion, we decided that inclusion of IL-3 and IL-6 gave no worthwhile advantage over SCF/Flt3-L/G-CSF alone for generation of neutrophil progenitors. TPO in combination with SCF/Flt3-L/G-CSF slightly reduced the expansion seen with SCF/Flt3-L/G-CSF alone, presented below.

### Phenotypes of expanded cells

**Figure 2:**
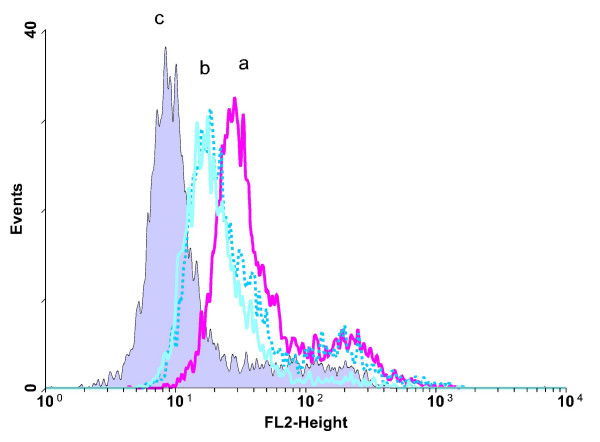
**Change in CD34 expression of cultured cells**. Anti-CD34-PE staining of 14-day cultured cells stimulated with 100 ng/ml SCF + 10 ng/ml Flt3-L with or without 100 ng/ml G-CSF. (a) SCF + Flt3-L alone; (b) SCF + Flt3-L with G-CSF (solid line = day 0; dotted line = day 7); (c) no anti-CD34, isotype control (for SCF + Flt3-L alone, but controls for the other cultures show overlapping peaks). Gated on intact cells (forward/side scatter) and CD45 staining.

After 14 days stimulation with SCF/Flt3-L, all cells stained with anti-CD34 (Figure 2a). The majority of cells stained to medium fluorescence intensity, with a small population of CD34-bright cells. When G-CSF was added to SCF/Flt3-L at day 0 there was a dimming of the major population medium-fluorescence-intensity CD34-staining peak, and disappearance of the bright CD34^+ ^population (solid line, b). When G-CSF was added to SCF/Flt3-L at day 7 there was a similar dimming of the major population medium-fluorescence-intensity CD34-staining peak which appeared less dimmed, and retention of the bright CD34^+ ^population (dotted line, b), which implied the effect is less than addition of G-CSF at day 0. In no case were there any obvious surviving CD34-negative cells at 14 days, except when G-CSF was used alone without SCF/Flt3-L (not shown because of very low cell numbers, not suitable for histogram representation).

We used expression of CD16b as a non-labile neutrophil-specific marker. Cells cultured with SCF/Flt3-L/G-CSF showed a small shift up in CD16b staining compared to unstained and isotype controls or compared to cells cultured with SCF/Flt3-L alone, but all were dim compared to mature peripheral blood neutrophils which in our hands stain brightly with CD16b (not shown). There was a major shift up in intracytoplasmic myeloperoxidase expression in cells cultured with SCF/Flt3-L/G-CSF compared to cells cultured with SCF/Flt3-L alone (not shown).

### Morphology

CD34^+ ^cells expanded with SCF/Flt3-L alone and stained in Wright-Giemsa remained morphologically undifferentiated, with no clear indication of development of granulocytic morphology. When G-CSF was added there was an extensive development of neutrophil morphology. Segmented cells in different stages of neutrophil maturation were found and around 80% of the cells were in a quite mature stage with apparent nuclei segmentation, lobulation, and granularity in the cytoplasm (see below under *Effects of TPO *where these studies were repeated with/without addition of TPO).

### Colony assays

Methocult colony assays were carried out for some PBSC blood samples on cells before culture (day 0) and at day 14 following cytokine expansion culture (Table 1). Because of limited cell numbers following CD34^+ ^cell enrichment, phenotype, but not CFC assays, were carried out on CD34^+ ^enriched cells before culture. Instead pre-expansion CFC assays were performed on the mononuclear cells before CD34^+ ^enrichment. We therefore can only directly judge the qualitative results (CFC frequencies), but have included available data as some indirect quantitative guidance. Before expansion, the predominant colonies generated were erythroid BFU-E, but after culture with SCF/Flt3-L or SCF/Flt3-L + G-CSF the cells showed a pronounced shift to myeloid commitment. There was a marked shift towards CFU-GM and away from BFU-E in all expanded cultures. CFU-GM were consistently higher and BFU-E lower in cultures with G-CSF compared to SCF/Flt3-L alone, but the switch to CFU-GM with SCF/Flt3-L alone compared to unexpanded cells is almost as large as that with SCF/Flt3-L + G-CSF, and may indicate that most expanded cells are myeloid committed, even without addition of G-CSF.

**Table 1:** Colony formation by day 0 mononuclear cells and by day 14 expanded CD34^+ ^cells.

	% BFU-E	% CFU-GM + % CFU-G	BFU-E per thousand cells	CFU-GM per thousand cells	% CD34 in CFC assay	CD34 fold expansion in culture
Day 0						
exp 28	74	26	35.9	12.4	2.6	
exp 29	74	26	8.6	3.0	1.2	
exp 32	72	28	5.0	1.9	0.4	
						
Day 14						
exp 28 SF	10	90	3.0	28.5	100	17.5
exp 28 SFG	5	95	0.8	16.1	100	33.4
						
exp 29 SF	18	82	1.1	4.9	100	1.8
exp 29 SFG	9	91	0.4	4.5	100	2.8
						
exp 32 SF	13	87	0.67	4.7	100	10.8
exp 32 SFG	0	100	0	4.7	100	32.4

CFC comparisons in 3 sets of mobilised PBSC before culture (day 0: mononuclear cell isolates, not CD34+ enriched) and after culture (day 14: expanded CD34+ enriched cells) with SCF/Flt3-L (SF) and with SCF/Flt3-L + G-CSF (SFG). The proportional (percent) results were used for qualitative comparisons. Some indication of quantitative responses can be obtained from CFC numbers, which may relate to CD34+ numbers allowing for CD34+ expansion.

### Neutrophil superoxide activity

An important index of neutrophil function is the production of superoxide radicals upon activation, which we measured here by detecting luminol-amplified chemiluminescence responses to phorbol myristate acetate (PMA). We compared the respiratory burst activity of peripheral blood neutrophils from healthy adult donors with the respiratory burst activity of CD34^+ ^enriched mobilised PBSC expanded in SCF/Flt3-L alone or expanded in SCF/Flt3-L with G-CSF. CD34^+ ^PBSC cultured with SCF/Flt3-L alone show no bioluminescence response to PMA, whereas those cultured with SCF/Flt3-L and G-CSF respond like mature healthy neutrophils (see below under *Effects of TPO *where these studies were repeated with/without addition of TPO).

### Effects of TPO on optimal expansion and maturation of CD34^+ ^enriched mobilised PBSC

**Figure 3:**
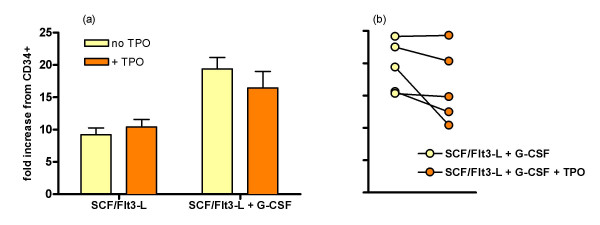
**Effect of TPO on expansion of CD34^+ ^cells from five patients**. (a) Effect of TPO on expansion in SCF + Flt3-L alone and in SCF + Flt3-L + G-CSF. Bars represent mean and standard error. (b) Individual patient's responses in SCF + Flt3-L + G-CSF with or without TPO. These are different patients from those shown in figure 1.

The addition of TPO to the above SCF/Flt3-L/G-CSF neutrophil expansion/differentiation cocktail was tested by examining cell expansion, morphology and function. The addition of TPO to SCF/Flt3-L showed no significant change in expansion compared to SCF/Flt3-L alone. The addition of TPO to SCF/Flt3-L/G-CSF slightly reduced or had no effect on the expansion with SCF/Flt3-L/G-CSF alone (Figure 3). This reduction did not reach significance (p = 0.0625, Wilcoxon signed rank test, n = 5) in the small number of paired samples tested, but may indicate a trend which would become significant in a larger sample. Certainly TPO offered no advantage over SCF/Flt3-L/G-CSF alone in terms of cell expansion, and could be omitted for the sake of culture simplicity on these grounds alone.

**Figure 4:**
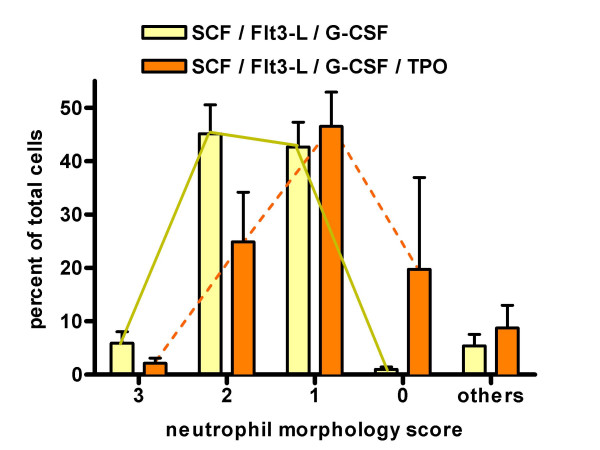
**Morphological changes in 14-day cultured cells from four patients stimulated with SCF/Flt3-L/G-CSF alone or SCF/Flt3-L/G-CSF + TPO**. Cells (200 per slide) were scored as 0 (no morphological change, resembling lymphocytes) and from 1 to 3 on degree of developing neutrophil morphology. Any cells with non-neutrophil morphological changes were classed as others. Bars represent mean and standard error; lines connect the scores for each cytokine cocktail so that curve shifts can be seen.

**Figure 5:**
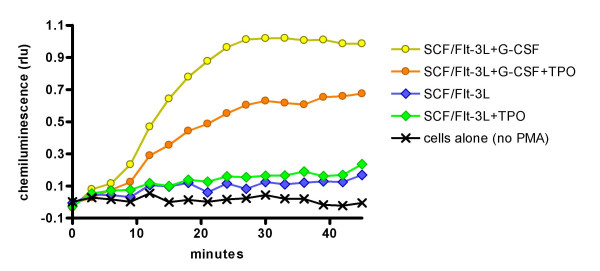
**PML-stimulated chemiluminescence responses of cells expanded with different cytokine combinations**. PML-stimulated chemiluminescence responses of an individual patient's 14-day cultured cells stimulated with combinations of SCF + Flt3-L with and without G-CSF and the effect of TPO on these combinations. The baseline (no PMA) is for the SCF/Flt3-L/G-CSF combination: the baselines for the other cytokine combinations overlaid this baseline and are not shown.

Cells expressing CD41 were found in small numbers in cultures to which TPO was added, but were not found in any cultures when TPO was omitted (not shown). When cells were counted and scored according to their degree of neutrophil differentiation, cells stimulated with SCF/Flt3-L/G-CSF + TPO were less differentiated with less mature neutrophil morphology than cells cultured with SCF/Flt3-L/G-CSF alone (Figure 4). Myeloperoxidase (MPO) activity is found in mature neutrophils and myelocyte progenitors. The CD34^+ ^cells cultured with SCF/Flt3-L/G-CSF with TPO show a marginal decline in intensity of expression of MPO compared with the cells cultured without TPO (not shown). However, since MPO stains most of the stages of the committed neutrophil differentiation pathway then an effect by TPO on the stage of neutrophil maturation achieved might not be clearly detected with this method. Neutrophil superoxide activity is a good indicator of neutrophil functional maturity. CD34^+ ^cells cultured with SCF/Flt3-L/G-CSF show a maximal superoxide response to PMA, similar to fresh mature peripheral blood neutrophils. Cells cultured with SCF/Flt3-L without G-CSF show little superoxide response whether or not they are cultured with TPO. When TPO is added to CD34^+ ^cells cultured with SCF/Flt3-L/G-CSF, the superoxide response is reduced, but not abolished (Figure 5). In this respect, TPO appears antagonistic to the effect of G-CSF on promoting maturation of neutrophil superoxide activity in the expanded CD34^+ ^cells.

## Discussion

These studies indicate that SCF and Flt3-L synergise to provide a considerable proliferative stimulus to expand PBSC CD34^+ ^cells in culture, which is not significantly increased by supplementary addition of IL-3, IL-6 or TPO alone or in combination. Addition of G-CSF to SCF and Flt3-L further expands total PBSC CD34^+ ^cells and drives neutrophil differentiation and maturation. The resultant cells have reduced expression of CD34, express neutrophil morphology, and acquire neutrophil function as assessed by myeloperoxidase activity and superoxide generation in response to PMA. Supplementary addition of TPO to SCF/Flt3-L/G-CSF reduces maturation of neutrophil morphology and function, and has a slight inhibitory effect on total CD34^+ ^cell expansion which does not reach significance. This indicates that for selective *ex vivo *expansion of neutrophil precursors from PBSC, it is valuable to include Flt3-L with SCF and G-CSF for maximal expansion, and that supplementary inclusion of IL-3 and/or IL-6 is superfluous and increases the complexity and cost of protocols. Supplementary inclusion of TPO, favoured in protocols aimed to produce combined expansion of both neutrophil and megakaryocyte precursors, shows no benefit in terms of expansion and retards the maturation of neutrophil precursors.

A variety of different studies have examined HSC expansion *ex vivo *and have used a variety of parameters to measure outcomes. Because of limited sample volumes we preferred to measure quantitative outcomes by cell counting and morphological and phenotype analysis, and clonogenic colony assays were only carried out on a small number of samples, comparing day 0 colony assays on PBSC mononuclear cells before enrichment of CD34^+ ^cells with day 14 assays on cells cultured after CD34^+ ^enrichment. While the results expressed in Table 1 are indicative of CFU expansion, they are indeterminate quantitatively and were used only for qualitative comparison. Our main quantitative method is based on cell counts and phenotype and morphology measurements as used in some reported *ex vivo *PBSC expansion studies[25,26] and also as used in a study of neutrophil expansion from cord blood[27]. Expression of neutrophil enzyme activities and functional activity is also used in two of these studies[27,25]. In our study colony assays showed slight differences between cells expanded with SCF/Flt3-L and cells expanded with SCF/Flt3-L/G-CSF in that more BFU-E colonies were retained in the absence of G-CSF, but myeloid colonies predominated in both expansion cultures whereas erythroid colonies predominate before expansion, as has been reported by Reichle et al[14]. Thus colony assays could not be used to sensitively assess the effects of other supplementary cytokines on SCF/Flt3-L/G-CSF. We did not use CD16 in this study since it is not neutrophil specific and our observations indicate that it is rapidly lost from mature neutrophils. Expression of CD16b in expanded cells was dim compared to mature neutrophils and differences in expression between SCF/Flt3-L and SCF/Flt3-L/G-CSF expanded cells were marginal and were not used to evaluate effects of supplementary cytokines. For expression of superoxide production addition of G-CSF to SCF/Flt3-L is necessary. This showed a marked reduction when TPO was added to the SCF/Flt3-L/G-CSF combination. Addition of TPO also shifted the morphology to a less differentiated profile.

From the outset of *ex vivo *expansion Haylock et al[28] promoted autologous PBSC CD34^+ ^cell expansion for reduction of neutropenia as an adjunct to unmanipulated PBSC engraftment. Many subsequent studies have targeted expansion of all classes of HSC, including primitive long-term reconstituting cells and mature lineage-committed progenitors, to provide sufficient reconstitutive graft materiel from smaller starting amounts of PBSC, without support from unmanipulated PBSC. However, Holyoake et al[29] demonstrated clinically that transplantation solely with *ex vivo *expanded CD34^+ ^cells does not confer durable haematopoietic reconstitution, and that unmanipulated PBSC are required for durable reconstitution. Evidently it is difficult to achieve simultaneous expansion of committed progenitors and preservation or expansion of more primitive multipotent HSC in the same system. Of necessity this remains the target of advocates of the use of umbilical cord blood HSC for allogeneic transplantation where autologous PBSC transplantation is not an option, since although cord blood is rich in CD34^+ ^cells the volumes are limited and absolute CD34^+ ^cell amounts are low compared to that available from PBSC by apheresis collection[30]. As has recently been reviewed[31] the goal of many cord blood expansion protocols remains a single complex expansion system delivering mature trilineage erythroid, myeloid and megakaryocyte precursors for rapid reconstitution with retention of multipotent stem cells for durable reconstitution. Some cord blood studies promote the use of multiple cords, some of which may be manipulated *ex vivo *for selective lineage expansion[32-34]. The lack of durable reconstitution by expanded cord blood cells alone has been confirmed in animal studies[35], and indicates the necessity of ensuring retention of multipotent long-term reconstituting HSC, for which single system protocols are still being sought[36].

Published PBSC expansion protocols all employ SCF as the major direct proliferative stimulus for primitive and committed HSC. Other cytokines are used with SCF to provide additional direct stimuli, or synergistic stimuli, for proliferative expansion. Early preclinical studies of *ex vivo *expansion of PBSC CD34^+ ^cells employed highly complex cytokine mixtures[28,37]. However, Flt3-L was not available to these investigators, and it appears that it may substitute most other cytokine mixtures in synergising with SCF for HSC expansion. Further cytokines are employed to stimulate mature and immature HSC to differentiate and mature along specific lineage-committed progenitor pathways, and may also provide proliferative and survival signals for these committed cells. It has been shown that certain combinations of cytokines can produce expansion of both primitive and committed haematopoietic progenitors from PBSC in the same system[38]. It may be naïve to assume that all of these processes can proceed simultaneously in the same system without competition or inhibition between different HSC expansion and differentiation pathways. High level of complexity of cytokine cocktails may be required to achieve multilineage expansion yet retain primitive multipotent HSC for long term reconstitution, which is the objective for cord blood. This is probably not required for selective neutrophil expansion only, as an adjunctive support for unmanipulated autologous PBSC transplantation.

For PBSC the focus has largely turned to expansion of lineage-committed progenitors for more rapid reconstitution, to be given as an adjunct to unmanipulated PBSC which supply durable reconstitution. Most of the published protocols have targeted simultaneous expansion of both neutrophil and megakaryocyte precursors, and many have simplified the expansion protocols for clinical application to include only SCF, G-CSF and TPO. Most of these have reported significant clinical reduction of neutropenia when these expanded cells are given with unmanipulated PBSC compared with unmanipulated PBSC alone[15,11,10,13], and in some cases neutropenia was virtually abolished[15,11]. Recent studies of PBSC expansion by SCF, G-CSF and TPO in new medium formulations have shown retention of long-term reconstituting HSC activity *in vitro*[39], and as well as abrogating neutropenia when used as an adjunct to unmanipulated PBSC these have demonstrated durable clinical haematopoietic reconstitution by expanded cells alone, not as an adjunct to unmanipulated PBSC[11].

The outcome of using adjunctive PBSC expanded with multilineage protocols on clinical reduction of thrombocytopenia ranged from no effect[10,15] to a reduction in post-transplant platelet transfusion requirement[13,11] or a mean reduction in thrombocytopenia duration by 1 day[12]. More complex multilineage expansion protocols such as those used for cord blood do not appear to have been evaluated clinically. Selective *ex vivo *expansion of megakaryocyte precursors by relatively simple protocols including SCF, TPO and IL-3[22], or SCF and TPO only[21], showed no clinical benefit on thrombocytopenia reduction when compared to unmanipulated PBSC alone when administered as an adjunct to unmanipulated autologous PBSC. It therefore appears that thus far, for autologous PBSC transplantation, clinical neutropenia but not thrombocytopenia is amenable to reduction by simple cytokine *ex vivo *expansion protocols.

A few studies have investigated selective *ex vivo *expansion exclusively of neutrophil progenitors from PBSC[26,40,25,14] and in two such studies the expanded cells have been used clinically to supplement autologous PBSC. Results were inconclusive in one clinical study[40] but reduced neutropenia in the other[14]. These studies employed a variety of expansion protocols, culture mediums and *in vitro *outcome assessments, and it is difficult to directly compare results quantitatively or qualitatively when described by clonogenic colony numbers or by different total or phenotypically or morphologically distinct cells numbers. However assessed, all agree that it is possible to expand neutrophil precursors *ex vivo *from PBSC.

Our results are consistent with the findings of others that TPO is not necessary for selective neutrophil expansion from PBSC[25,14,26,40] or cord blood[27] CD34^+ ^cells. Its inhibitory effect on neutrophil maturation is probably marginal, but it is certainly superfluous for specific neutrophil precursor expansion. Consistent with our *in vitro *observations, it has been reported that *in vivo *administration of TPO delays myeloid recovery following chemotherapy[41]. Others have suggested that megakaryocyte expansion may be best achieved with quite different cytokine combinations from those required for neutrophil precursor expansion[20,42,43]. It may be best to focus on selective neutrophil precursor expansion as a feasible and needed goal for reduction of neutropenia, and if megakaryocyte generation is to be considered then separate protocols should be devised to optimise this in distinct cultures rather than attempting simultaneous neutrophil and megakaryocyte generation in the same system. These results in preliminary small-scale laboratory culture suggest that the combination of SCF, Flt3-L and G-CSF should be investigated for development of protocols suitable for clinical scale-up and GMP compliance, as a possible cost-effective means of reducing neutropenia following autologous PBSC transplant which is not addressed by availability of donor granulocyte transfusion.

## Conclusion

We have systematically investigated a number of combinations of cytokines for *in vitro *culture expansion of CD34^+ ^cells from G-CSF mobilised PBSC, and have found that a combination of SCF, Flt3-L and G-CSF synergise to give maximal expansion and neutrophil maturation. Addition of other cytokines commonly employed for *ex vivo *PBSC expansion did not increase cell numbers or improve neutrophil maturation, and appear superfluous. Addition of TPO, commonly employed in multilineage PBSC expansion to enhance megakaryocyte production, appeared to have a moderate retarding effect on both expansion and maturation of neutrophils from CD34^+ ^cells, so that TPO appears at best superfluous and at worst detrimental to *ex vivo *neutrophil expansion by SCF, Flt3-L and G-CSF. While platelet transfusion is standard but few transfusion centres offer granulocyte transfusion, and while multilineage expansion does not reduce thrombocytopenia, we recommend that *ex vivo *neutrophil expansion from PBSC by a neutrophil specific expansion protocol such as that above should be investigated as an adjunct to autologous PBSC transplantation for reduction or abrogation of post-transplant neutropenia.

## Competing interests

The author(s) declare that they have no competing interests.

## Authors' contributions

JD and MLT conceived of the study and attracted funding.

GRB and OT designed, coordinated and analysed the study, and drafted the manuscript.

HR, JD and MLT authorized access to patient and blood donor samples for the study and contributed clinical expertise and oversight to the study design.

OT carried out cell isolation and culture, FACS and functional analysis as a component of her PhD thesis studies under the supervision of GRB.

All authors read and approved the final manuscript.
